# Advancements in Targeting Ion Channels for the Treatment of Neurodegenerative Diseases

**DOI:** 10.3390/ph17111462

**Published:** 2024-10-31

**Authors:** Yuxuan Li, Jingxuan Fu, Hui Wang

**Affiliations:** 1 Key Laboratory of Molecular Biophysics of Hebei Province, Institute of Biophysics, School of Health Sciences and Biomedical Engineering, Hebei University of Technology, Tianjin 300401, China; 202221103004@stu.hebut.edu.cn (Y.L.); fujingxuan@hebut.edu.cn (J.F.); 2 School of Chemical Engineering and Technology, Hebei University of Technology, Tianjin 300401, China

**Keywords:** ion channels, neurodegenerative disease, Alzheimer’s disease, Parkinson’s disease, Huntington’s disease, amyotrophic lateral sclerosis, multiple sclerosis

## Abstract

Ion channels are integral membrane proteins embedded in biological membranes, and they comprise specific proteins that control the flow of ion transporters in and out of cells, playing crucial roles in the biological functions of different cells. They maintain the homeostasis of water and ion metabolism by facilitating ion transport and participate in the physiological processes of neurons and glial cells by regulating signaling pathways. Neurodegenerative diseases are a group of disorders characterized by the progressive loss of neurons in the central nervous system (CNS) or peripheral nervous system (PNS). Despite significant progress in understanding the pathophysiological processes of various neurological diseases in recent years, effective treatments for mitigating the damage caused by these diseases remain inadequate. Increasing evidence suggests that ion channels are closely associated with neuroinflammation; oxidative stress; and the characteristic proteins in neurodegenerative diseases, including Alzheimer’s disease (AD), Parkinson’s disease (PD), Huntington’s disease (HD), amyotrophic lateral sclerosis (ALS), and multiple sclerosis (MS). Therefore, studying the pathogenic mechanisms closely related to ion channels in neurodegenerative diseases can help identify more effective therapeutic targets for treating neurodegenerative diseases. Here, we discuss the progress of research on ion channels in different neurodegenerative diseases and emphasize the feasibility and potential of treating such diseases from the perspective of ion channels.

## 1. Introduction

### 1.1. Classification of Ion Channels

Ion channels are widely distributed across biological membranes. In the context of the nervous system, they are integral to neurons, and they are responsible for initiating nerve impulses and synaptic transmission. Ion channels are classified into two categories: voltage-gated ion channels (such as Na^+^, K^+^, Ca^2+^, and Cl^−^) and ligand-gated ion channels (such as nicotinic acetylcholine receptors, GABA receptors (GABAB receptors act in conjunction with G protein-coupled receptors and do not exist directly as ion channels), and NMDA receptors). These ion channels collectively participate in the process of synaptic transmission [1].

These ion channels are involved in various physiological cell activities, such as cell migration, cell communication, or cell adhesion. With respect to the physiological activities that maintain brain homeostasis, ion channels can transmit electrical signals in order to receive and accompany information transmission [2]. Previous studies have demonstrated that genetic defects or abnormalities in the genes responsible for these channels can result in a range of nervous system diseases, such as memory disorders, motor impairments, and neuromuscular conditions [3]. Neurodegenerative ion channel diseases, such as myotonia, congenital myasthenia syndrome, malignant hyperthermia, and periodic paralysis, are caused by ion channel defects. Similarly, a variety of neurodegenerative diseases also involve ion channel defects, although the underlying mechanisms are not yet fully understood. Furthermore, numerous channel-specific inhibitors and activators have been employed in scientific investigations and therapeutic interventions for various diseases. There is potential for these agents to be utilized in the treatment of related neurodegenerative conditions. Currently, it is widely accepted that ion channels play the following roles in regulating membrane physiology [1]: To establish the membrane potential of the cell, the sodium ion channel rapidly opens at the onset of the action potential, resulting in the depolarization of the cell membrane as the potassium ion channel closes. Subsequently, the sodium ion channel closes, and the calcium ion channel opens to sustain the depolarized state. Once the calcium ion channel becomes inactivated, the potassium ion channel opens, and membrane repolarization occurs [4]. Ion channels also act as regulators and regulate the cell’s volume, which is realized by maintaining the cell membrane’s internal and external electrolyte balance [5]. Physiological processes such as neurotransmitter release and muscle contraction can also be controlled via ion channels that regulate electrical signals within cells [6].

### 1.2. Neurodegenerative Diseases

Alzheimer’s disease (AD), Parkinson’s disease (PD), Huntington’s disease (HD), multiple sclerosis (MS), and amyotrophic lateral sclerosis (ALS) are all classified as neurodegenerative diseases. Neurodegenerative diseases are characterized by the degeneration of the peripheral or central nervous system, and there is currently no cure for these diseases [7]. The hyperphosphorylation of tau proteins, mutation of the catalytic domain of γ-secretase, and accumulation of Aβ are the main causes of AD. AD can cause neuronal [8]. Oxidative stress and mitochondrial damage, as well as environmental factors, can cause PD, resulting in slow movement [9]. The HTT (Huntingtin) protein is a protein that is widely expressed and located in the human body, with a gene named HTT, located on human chromosome 4 [10]. The abnormal amplification of the HTT gene results in the development of HD. Mutated HTT proteins accumulate in cells, resulting in lesions and symptoms such as mood swings and uncontrollable movements, which are symptoms of HD [11]. Multiple sclerosis (MS) is a chronic degenerative disease that manifests as speech, muscle, and visual dysfunction. It is caused by demyelinating plaques that form in the brain and spinal cord, which result in inflammatory neuronal damage [12]. Amyotrophic lateral sclerosis (ALS) is also a chronic motor neuron degenerative disease. The most prominent feature is motor dysfunction, with neuronal loss occurring in many areas of the brain. The mortality rate is significant [13]. Different diseases affect daily lives to a certain extent. Therefore, the search for neurodegenerative disease treatments remains an ongoing and pressing challenge.

### 1.3. The Potential Role of Ion Channels in Neurodegenerative Diseases

Ion channel proteins facilitate the passage of sodium, potassium, calcium, and chloride ions through the lipid bilayer, which is generally impermeable. They also play a crucial role in regulating various physiological processes, such as the electrical conduction of nerve cells, muscle contraction, and the release of neurotransmitters.

**Figure 1:**
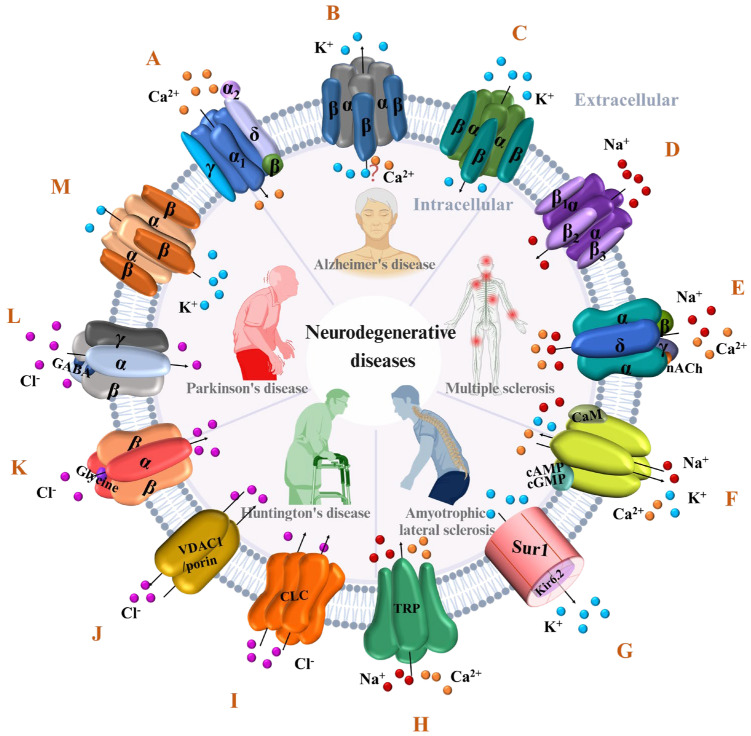
The major ion channels associated with neurodegenerative diseases: (**A**) voltage-gated calcium channel, (**B**) calcium-activated potassium channel, (**C**) inward-rectifier potassium channel, (**D**) voltage-gated sodium channel, (**E**) nicotinic acetylcholine receptor, (**F**) cyclic nucleotide-gated ion channel, (**G**) ATP-sensitive potassium channel, (**H**) transient receptor potential channel, (**I**) voltage-gated chloride channel, (**J**) mitochondrial voltage-dependent anion channel-1, (**K**) glycine receptor, (**L**) γ-amino butyric acid receptor, (**M**) voltage-gated potassium channel.

Ion channels play a pivotal role in regulating the influx and efflux of ions within the brain, contributing to neuronal growth, synaptic plasticity, neurotransmitter release, and neuronal motor excitability [2]. They also help maintain ion balance in the cell membranes of postsynaptic neurons and express specific channels. Furthermore, ion channels control the input to body fluids and synapses, collectively contributing to the physiological equilibrium of the brain. Their activity is often modulated by hormones, and neurotransmitters are regulated by the metabolic center of the brain [14]. During cerebral hypoxia, glutamate, an excitatory neurotransmitter, induces the influx of cations through ion channels, resulting in excitatory toxicity due to depolarization caused by hypoxia [15,16]. In ischemia, the excessive release of glutamate from the presynaptic terminal results in the overactivation of glutamate receptors (NMDA (N-methyl-D-aspartate) and AMPA (α-amino-3-hydroxy-5-methyl-4-isoxazole propionic acid) receptors), resulting in an excessive influx of calcium ions. NMDA receptors form a heterotrimeric complex with Panx1 and the tyrosine kinase Src [17], and they activate pannexin channels, hyperactivating them and releasing ATPs into the extracellular space. The buildup of extracellular glutamate and ATPs continuously activates various complexes, potentially triggering a cascade of apoptosis and necrosis and ultimately resulting in neuronal damage or death [18]. In the central nervous system, ion channels are crucial in controlling cell migration and regulating signaling processes [19]. Mutant sodium, potassium, and calcium ion channels, as well as acetylcholine- and glycine-gated channels, have been implicated in conditions such as convulsions, epilepsy, myasthenia gravis, Alzheimer’s disease, schizophrenia, and Parkinson’s disease [20]. Many ion channels are implicated in neurodegenerative diseases, influencing disease onset and progression (Figure 1). Given their impact on neurodegenerative diseases, the study of ion channels presents a promising avenue for addressing these conditions. Thus, this article has a strong focus and therapeutic potential compared to other articles; we focus almost exclusively on potential drugs that target ion channels for treating NDDs rather than targeting pathway proteins or other targets.

## 2. Ion Channels in Neurodegenerative Diseases

### 2.1. Alzheimer’s Disease

In addition to the K^+^ and Ca^2+^ channels, which are the main ion channels affecting AD and playing a key role in the disease, some ion channels play a secondary role in the disease, such as RyR channels (ryanodine receptor channels), Cl^−^ channels (chloride channels), nAChR channels (neuronal nicotinic acetylcholine receptor channels), TRP channels (transient receptor potential channels), and NMDAR channels (N-methyl-D-aspartate receptor channels). A major factor in Alzheimer’s disease is the buildup of amyloid-beta (Aβ). Potassium ion channels (K^+^ channels) are essential for creating and sustaining neuronal potentials. Any disruption of these channels can lead to impaired neurotransmission and neuronal injury. The accumulation of Aβ in hippocampal neurons [21] inhibits the swift inactivation of K^+^ currents. Additionally, certain types of K^+^ channels have been associated with neurodegenerative changes via the activation of microglia in response to neuroinflammation triggered by toxic Aβ. In addition, it is reported that voltage-gated potassium channels, such as Kv1.5, and calcium-activated potassium channels, such as KCa3.1, can activate microglia [22]. In addition, Kv1.3 and KCa3.1 (gene symbol KCNN4) can also trigger neurodegenerative processes and cause Aβ-promoted neuro-inflammatory phenomena. Studies have shown that the K^+^ channels of fibroblasts in AD patients are not sensitive to 113-pS TEA (113-pS tetraethylammonium), which is a potassium channel blocker, unlike those of normal people [23]. This results in neuronal death due to excessive intracellular Ca^2+^ caused by insufficient K^+^ channel activity [24]. The reduced expression of the Kv3 subfamily has also been observed in AD [25], which impairs the action of Kv3 in promoting action potential repolarization [26]. In the human brain, Kv3 channels are rapidly activated at high-threshold voltages and allow a rapid efflux of potassium ions, which rapidly reduces the intracellular potential and accelerates the period of decline in the action potential, which is particularly important for high-frequency neuronal firing [27].

Intracellular calcium ions are stored in mitochondria. Calcium ions are also common secondary messengers that regulate various physiological processes of cells, such as proliferation, metabolism, and apoptosis. Once mitochondria store excessive calcium ions, it can cause apoptosis and abnormalities in mitochondrion-related pathways such as caspase activation. Therefore, abnormal calcium signaling can significantly cause mitochondrial dysfunction in cells. Similarly, it can be concluded that maintaining a stable calcium ion level is particularly important in neuroinflammation and oxidative stress [28]. In addition to mitochondria [29], the key regulators of free calcium ion concentrations in cells include the endoplasmic reticulum [30], lysosomes [31], and calcium-binding proteins [32], as well as calcium ion channels [33], calcium ATPases [34,35], and exchangers [36,37]. Hardy and Higgins were the first to report that the Aβ peptide disrupts the Ca^2+^ balance in neurons, resulting in an increase in intracellular Ca^2+^. This statement was later confirmed [38]. Recent research on Aβ-related ion channels has shown that Aβ peptides form abnormal ion channels in the neuronal membrane, disrupting the neuronal balance and thus being toxic to neurons. In AD patients, the balance of intracellular and extracellular Ca^2+^ is disrupted, affecting the function of glutamate receptors, glucose transport proteins, and the ion motive ATPase [39]. It has also been observed that the function of some voltage-gated calcium channels [40,41,42] and ligand-gated calcium channels [43,44,45] can be abnormally disrupted in AD patients. This disruption may be caused by the excessive accumulation of Aβ. Recent studies have shown that Ca^2+^ homeostasis in the TRP channel of AD patients is disrupted, affecting autophagy and tau metabolism, which exacerbates the symptoms of AD. In mammals, TRP channels behave as non-selective cation-permeable channels and are divided into 28 superfamilies or 6 subfamilies: TRPC1-7, TRPM1-8, TRPV1-6, TRPP1-5, TRPA1, and TRPML1-3 [46]. This effect is likely to start with the processing of the upstream β-amyloid precursor protein (AβPP) [47]. The hyperexcitable response of the hippocampus and cortex in AD patients may be caused by elevated Na^+^/K^+^ ATPase activity and L-type Ca^2+^ channels in the pathogenic region [48]. Elevated Na^+^/K^+^ ATPase activity may be a cellular compensatory response to Aβ toxicity in an attempt to maintain ionic homeostasis. Overactive Na^+^/K^+^ ATPase may lead to an increase in the intracellular calcium ion concentration because Na^+^/K^+^ ATPase activity is associated with the Na^+^/Ca^2+^ exchanger, which promotes Ca^2+^ endocytosis along with Na^+^ efflux [49]. Aβ can increase the frequency and duration of the opening of L-type Ca^2+^ channels, leading to intracellular calcium overload. Chronic calcium overload also impairs synaptic function and neuroplasticity, which is associated with cognitive decline in AD [50]. It has been suggested that the Aβ peptide binds into small aggregates that form Ca^2+^-permeable channels in the plasma membrane of neurons, causing the process of Ca^2+^ endocytosis [51].

In addition to the above-mentioned primary ion channels, recent studies have uncovered the presence of intracellular chloride ion channel 1 (CLIC1), situated on the plasma membrane of hippocampus-activated microglia in individuals with mild Alzheimer’s disease. Upon the Aβ stimulation of microglia, CLIC1 channels are significantly expressed, resulting in modifications in the membrane anion permeability of neurons and ultimately resulting in neuronal death [52]. Furthermore, the nAChR is implicated in the pathology of AD, as cholinergic depletion may elevate Aβ production and its neurotoxicity by altering the signal conduction event (reduced acetylcholine synthesis caused by reduced choline acetyltransferase activity, which in turn reduces cholinergic receptor activation, ultimately leading to a reduction in cAMP and a decrease in protein kinase activity) associated with cholinergic neurotransmission [53]. The high expression of nAChR subtypes α7 and α4β2 in regions affected by AD indicates their close relationship with the onset of AD. The reduction in cortical acetylcholine levels correlates strongly with the severity of AD symptoms [54].

### 2.2. Parkinson’s Disease

The pathogenic mechanism of Parkinson’s disease is intricate and multifaceted. There are numerous theories hypothesizing what contributes to Parkinson’s disease, including Ca^2+^ excess, mitochondrial malfunction, and oxidative or metabolic stress. The characteristics of dopaminergic neurons in PD patients are unstable membrane potential and the generation of intrinsic AP (action potential) [55]. The coordinated action of a multitude of voltage-gated ion channels expressed in the growing dendritic compartment exhibits spontaneous discharge activity [56], necessitating an active transport system and significant ATP consumption to continuously restore the ion gradient in the plasma membrane [57]. ATP levels must be continuously replenished through oxidative phosphorylation (OXPHOS) in the mitochondria, which is also the primary source of reactive oxygen species (ROS) in cells. Oxidative stress arises when the production of reactive oxygen species surpasses the capacity of the redox system to eliminate them. This stress induces non-specific chemical damage to cellular components, such as membrane lipids, proteins, and nucleic acids, resulting in potentially fatal consequences for cells [58]. In the context of Parkinson’s disease, several ion channels, including the Ca^2+^, K^+^, NMDAR, and Hv1 channels (voltage-gated proton channel 1), assume pivotal roles. Even minor irregularities in these ion channels can disrupt neuronal function. Abnormalities in voltage-gated T-type Ca^2+^ channels (TTCCs), Ca^2+^-sensitive voltage-gated A-type K^+^ channels (calcium-sensitive voltage-gated rapidly activatable and inactivatable potassium channels), voltage-gated L-type Ca^2+^ channels (LTCCs), and ATP-sensitive K^+^ (K-ATP) channels can all result in reduced dopamine function in the substantia nigra. The malfunction of neurons in the basal ganglia, particularly the dopamine (DA) neurons, triggers a progressive decline in neuronal activity, ultimately contributing to the onset of PD [59]. Furthermore, LTCCs act as autonomous pacemakers for neurons, and they are responsible for maintaining basic DA tension in target areas such as the striatum. Intracellular Ca^2+^ levels increase continuously as a result of the activation of Cav1.2 and Cav1.3 (Cav1.2 and Cav1.3 are both members of the LTCC family with similar structural and functional properties, but differ in their expression patterns and physiological roles) in the substantia nigra pars compacta (SNc) area during the pacing activity of dopaminergic neurons. This elevation triggers oxidation and an increase in mitochondrial stress, ultimately contributing to the potential death of neuronal cells.

Furthermore, the proteins Sur1 and Kir6.2, which are ATP-sensitive potassium channels, are highly expressed in dopaminergic neurons in the SNc and are connected to excitotoxicity, a condition that is intimately linked to Parkinson’s disease [60]. The G protein-gated inwardly rectifying IK1 channel Kir3.2 is non-selective when mutated, resulting in a significant increase in Na^+^ ions rather than K^+^ ions in cells. This causes the death of dopaminergic neurons in the SNc area and cerebellar cells [61]. Furthermore, the activity of K-ATP channels, in addition to specific subpopulations of dopaminergic neurons in the medial nigra region, promotes the shift from tonic discharge to NMDAR-mediated burst discharge, resulting in the temporal release of dopamine. Ca^2+^ can enter the cell when glutamate binds to the receptor and opens the NMDAR channels. Any change in glutamate transmission in Parkinson’s disease patients might cause dyskinesia. Ca^2+^ can enter the cell when glutamate binds to the receptor and opens the NMDAR channels. Any change in glutamate transmissions in Parkinson’s disease patients might cause dyskinesia [62]. Research has shown that Hv1 proton channels are present in human brain microglia [63] and immune tissues, and they play a crucial role in the generation of superoxide by NADPH oxidase during the respiratory burst of phagocytes, resulting in neurodegenerative damage [64].

### 2.3. Huntington’s Disease

Huntington’s disease (HD) is the most common monogenic neurodegenerative disease in Western countries [65]. In 1872, George Huntington reported a hereditary disease-like disorder with behavioral and neuropsychiatric manifestations that are almost completely explicit [66]. Huntingtin proteins are widely expressed in all animal and human cells and cause substantial disruptions in the brain, but their normal function is not yet clear [67]. Mutant Huntingtin proteins are prone to aggregation, and their pathogenicity may be related to the production of toxic mutant Huntington oligomers. This is due to the toxicity gain effect. The exact mechanism that causes neurodegenerative changes is not clear, and multiple processes are involved. Studies have shown that the amplification of the Huntingtin CAG repeat sequence may result in the abnormal splicing of the messenger RNA encoded by exon 1, resulting in the expression of toxic truncated proteins [68]. Under normal physiological conditions, Kir4.1 channels are essential for maintaining the resting membrane potential of cells and regulating extracellular K^+^ buffering in the brain. Research has shown that in striatal astrocytes expressing mHTT (mutant Huntington protein), there are alterations in Kir4.1 channel activity. This disrupts the balance of extracellular K^+^ levels, resulting in the heightened excitability of striatal neurons and the manifestation of HD motor symptoms. Additionally, other research groups have observed the reduced expression of K^+^ channel subunits (Kir2.1/Kir2.3) in striatal neurons in HD transgenic mouse models [69,70].

In addition to the abnormal function of K^+^ channels, various other ion channels (including Ca^2+^, Na^+^, and Cl^−^) have exhibited reduced expression in numerous studies involving HD mouse models. For example, it has been reported that mHTT in HD alters the function of the high-voltage-activation (HVA) Ca^2+^ channel [71]. In a separate study, the auxiliary subunit of the channel β4 subunit was found to be downregulated in both mouse models and the striatum of HD patients. The induced expression of β4 results in the growth of N2a (neuro-2a) cells in the hippocampus, indicating neuronal degeneration in transgenic mouse HD patients [72]. Additionally, in the R6/2 HD mouse model, a mouse model of HD with the addition of human Huntington gene fragments to the mouse genome [73], the expression of muscle ClC-1 chloride channels was also significantly reduced. As a result, functional alterations in these channels disturb the ion balance in cortical cone neurons; in the spinal cord, cortical pyramidal neurons transmit motor commands to the muscles via interneurons or motor neurons [74], thereby impacting neurotransmitter release, synaptic integration, and gene expression, all of which are crucial in causing cortical dysfunction in HD.

### 2.4. Amyotrophic Lateral Sclerosis

According to current research, the development of ALS involves factors such as glutamate excitotoxicity, oxidative stress, mutant superoxide dismutase 1 (SOD1) enzyme, mitochondrial dysfunction, and the disruption of axon transport processes [75]. Numerous studies have highlighted the involvement of multiple ion channels in ALS. For instance, the spontaneous activation of the voltage-gated Na^+^ channel (Nav1.5) is associated with the contraction of non-neural muscle fibers in mammals [76,77]. Moreover, a significant decrease in potassium ion channel (Kv1.2) expression has been observed in cases of human sporadic ALS [78]. The continuous conduction of Na^+^ ions followed by a sudden decrease in K^+^ ion conduction results in the overexcitation of axons, contributing to the symptoms of ALS [79].

The motor neurons that control the muscles in the tongue are susceptible to degenerative changes in ALS due to the differential expression of VGCCs (voltage-gated calcium channels). Studies have also demonstrated immune reactivity to various calcium ion channels (L-type, N-type (Cav2.2), P/Q-type (Purkinje cell/QT-type), and T-type) in ALS patients and animal models [80,81,82]. Israelson et al. investigated the role of mitochondrial ion channel disease in the progression of ALS and found that the mutant SOD1 inhibits mitochondrial voltage-dependent anion channel-1 (VDAC1/porin), resulting in mitochondrion-dependent apoptosis, which is fatal in ALS. However, further research is still needed to fully understand the mechanism of ion channel dysfunction in ALS [83].

### 2.5. Multiple Sclerosis

The key characteristic of multiple sclerosis (MS) is inflammatory neuronal damage caused by the presence of a large number of macrophages, T lymphocytes, microglia, and dendritic cells within the patient’s nervous system [12]. Neuronal damage in MS patients is primarily due to the infiltration of lymphocytes and macrophages, which make direct cell contact or release toxic substances such as glutamate or nitric oxide. They also indirectly cause damage through the loss of oligodendrocytes and myelin. In addition to inflammatory mediators, changes in electrical activity, intracellular Ca^2+^ overload, and mitochondrial dysfunction, along with subsequent neuronal death, are associated with the redistribution of certain voltage-gated and ligand-gated ion channels and transporters [84]. The altered expression patterns of specific voltage-gated sodium channel subtypes (both TTX-sensitive Na^+^ channels (Nav1.2, Nav1.5, and Nav1.6) and TTX-resistant Na+ channels (Nav1.8)) in multiple sclerosis play a role in axonal degeneration, resulting in subsequent cerebellar dysfunction [85,86]. In addition, these changes cause an influx of Na^+^ into the axon through the Nav channel, in turn activating the Na^+^/Ca^2+^ exchanger in reverse mode, leading to an influx of Ca^2+^ ions into the cell and a further increase in intracellular Ca^2+^ concentration via a calcium-induced calcium release mechanism and disrupting axon myelination [87], thus contributing to the pathogenesis of MS. Furthermore, the upregulation of calcium ion channels (Cav1.2, Cav1.3, Cav1.4, and Cav2.2) and potassium ion channel subtypes (Kv1.1, Kv1.2, Kv1.3, and Kv3.1 b (a splicing variant of the Kv3.1 channel)) hinders the conduction of demyelinated axons [88,89]. This elevation in calcium ion levels triggers apoptosis signals and leads to neuronal degeneration in MS.

The TRPM4 (transient receptor potential melastatin 4) channel is believed to be associated with multiple sclerosis as it is expressed during inflammatory lesions in the central nervous system [90]. Currently, there is no direct evidence of the abnormal function of chloride ion channels in MS patients. However, studies have noted that individuals with leukoencephalopathy [91] often exhibit defects in the autosomal recessive ClC-2 chloride ion channel, indicating a likely chloride (Cl^−^) involvement in MS.

## 3. Treatment Targeting Ion Channels

Neuroprotection is of utmost importance in addressing neurodegenerative diseases; however, our limited understanding of their underlying pathogenic mechanisms poses a challenge for effective treatment. Preclinical and genetic studies have uncovered the unexpected complexity and diversity of the molecular pathways involved in these diseases, while ongoing research, clinical approaches, and solutions are being pursued to address these conditions [92]. Based on the role of different ion channels in neurodegenerative diseases, methods for treating diseases comprise targeting ion channels and using ion channel modulators. Using ion channels as drug targets provides many advantages for the treatment of neurodegenerative diseases. For example, ion channels are usually widely expressed in vivo, and the structure of most ion channel analyses is relatively clear, which provides excellent conditions for the design of ion channel-targeted drugs. In addition, small molecules are powerful ligands for ion channels, and they are indispensable tools for researching [93] and treating ion channel-related diseases. Small-molecule drugs are easy to synthesize, and they respond quickly, providing convenient conditions for studying diseases.

### 3.1. Progress in the Treatment of AD

In the last two decades, there has been a significant increase in Alzheimer’s disease-related deaths [94]. The majority of drugs used for treatment are acetylcholinesterase inhibitors (AChEIs), such as donepezil, rivastigmine, tacrine, and galantamine, and N-methyl-D-aspartic acid receptor (NMDAR) antagonists, such as memantine [95]. Memantine acts as a low-affinity antagonist of extrasynaptic NMDA receptors, which are the primary mediators of excitotoxic components in neuronal degeneration, and it is believed to possess neuroprotective properties [96]. While some clinical studies have indicated that memantine has a certain impact on cognitive symptoms, the neuroprotective effect of memantine remains undetermined [97]. The intricate pathogenic mechanism in AD pathology is impeding the development of effective treatment strategies. In AD, three neuropathological processes occur: the formation of the extracellular matrix of Aβ plaques through the aggregation of insoluble Aβ oligomers, the formation of NFTs (neurofibrillary tangles) through the excessive phosphorylation of tau proteins, and the subsequent loss of neurons [98]. Some drugs have been unsuccessful in improving the cognition of patients with mild to moderate AD because they only target specific pathologies and do not address other neuropathological conditions [99]. Di Scala et al. demonstrated that Zn^2+^ significantly inhibits the intracellular Ca^2+^ elevation induced by Aβ_22–35_. The identification of linear-cholesterol-binding domains in Aβ offers a new therapeutic strategy for alleviating Aβ neurotoxicity [100]. Studies by Montecinos-Oliva et al. have shown that tetrahydrohyperforin (IDN5706) causes the neuroprotective effects of hippocampal brain tablets by activating the TRPC3/6/7 (transient receptor potential canonical 3/6/7) channel subfamily. The discovery of the mechanism of action of this drug is a necessary step for the clinical application of IDN5706 in Alzheimer’s disease [101]. Ruiqing Ni et al.’s studies revealed that the α7 nAChR agonist (methyllycaconitine, α-bungarotoxin, and mecamylamine) disrupts the interaction between fiber Aβ and α7 nAChR in the frontal cortex of the AD brain, potentially leading to the release of Aβ from the complex. Furthermore, α7 nAChR agonist therapy has the potential to regulate the pathogenic signaling mechanism of Aβ/α7 nAChR in the AD brain and may emerge as a novel approach for treating AD [102]. Li et al. demonstrated that icariin could inhibit the increase in abnormal intracellular calcium currents in hippocampal pyramidal neurons induced by Aβ_25–35_ in a dose-dependent manner. This suggests that icariin has the potential to protect neurons from abnormal intracellular calcium currents induced by Aβ, and it could be considered a potential drug candidate for treating patients with AD [103]. Additionally, Fernández-Morales et al. proposed a compound with three components: a dihydropyridine moiety (blocking the L channel and reducing the internal flow of calcium), a benzothiazide moiety (blocking the MNCX (mitochondrial Na^+^/Ca^2+^ exchanger) and slowing the outflow of calcium from the mitochondrial matrix into the cytoplasm), and a polyphenol moiety (to neutralize excess free radicals). This compound can eliminate pathologically enhanced NCC (neuronal Ca^2+^ cycling) and MCC (mitochondrial Ca^2+^ cycling), thereby delaying the onset of apoptosis and the death of vulnerable neurons. As such, this multifunctional compound has the potential to become a neuroprotective drug capable of slowing the progression of AD in patients [104] (Table 1).

### 3.2. Progress in the Treatment of PD

The theory that excitatory toxicity contributes to the development of Parkinson’s disease has been the basis for numerous preclinical and clinical studies. Memantine has been extensively studied in both preclinical models and clinical trials and is recognized as a neuroprotective agent for Parkinson’s disease and other neurodegenerative conditions. Clinical trials have exhibited moderate success in addressing the cognitive decline [105] and motor symptoms [106] associated with Parkinson’s disease. However, there is currently no literature supporting the use of memantine as a neuroprotective agent for patients with prodromal or early-stage Parkinsonism. Similarly, the voltage-gated sodium channel blocker Riluzole has been used to counteract the excitatory components of ALS and is currently being used in its treatment. Despite being tested as a neuroprotective agent in preclinical models of PD over the years, Riluzole’s efficacy is unsatisfactory [107]. Angelique Camilleri et al. tested six small-molecule compounds and black tea extracts to inhibit the ability of Aβ_42,_ alpha-synuclein, and tau aggregation complexes in penetrating the mitochondrial membrane. They found that the black tea extract and rosmarinic acid are the most effective mitochondrial protectants, suggesting that they may alleviate mitochondrial dysfunction in neurodegenerative diseases [108]. Quik et al. demonstrated that the activation of nAChRs initially triggers various intracellular signaling pathways primarily by altering calcium signals. When acetylcholine binds to nAChRs, the channel opens, allowing an influx of cations (mainly Na^+^ and Ca^2+^) into the cell. The influx of Na^+^ leads to depolarization of the cell membrane, which can further activate the VGCCs, in particular the L-type calcium channels. The activation of these channels leads to more Ca^2+^ influx into the cell [109]. As a result, the immune response and adjustments in nutritional factors may ultimately contribute to nicotine’s ability to reduce or prevent the neuronal damage observed in PD. Nicotine binds to the α-subunit of nAChRs, altering its conformation to allow cations to pass through. Apart from its potential neuroprotective effects, nicotine also exhibits antidepressant properties and enhances attention and cognition [110]. Beate Ritz et al. found that centrally acting L-type calcium channel blockers, such as nimodipine and nitrendipine, have the potential to provide neuroprotection in PD by preventing cytoplasmic DA-induced SNc neuronal cell death [111]. Furthermore, studies carried out by Li-Fang Hu et al. have indicated that iptakalim may regulate glutamate transporters by opening the mitoKATP channel (mitochondrial ATP-sensitive potassium channel), thereby reducing MPP^+^ (1-methyl-4-phenylpyridinium)-induced extracellular glutamate levels and protecting SH-SY5Y cells from MPP^+^-induced cytotoxic effects [112] (Table 2).

### 3.3. Progress in the Treatment of ALS, HD, and MS

For ALS, increased neuronal excitability and excitotoxicity are the main determinants of amyotrophic lateral sclerosis (ALS), also known as Lou Gehrig’s disease, and these observations have spurred efforts to develop neuroprotective treatments based on a fundamental principle. Currently, the only approved drug for ALS treatment is Riluzole, which is believed to exert its neuroprotective activity by inhibiting the Nav1.6 channel and excessive glutamate release [113]. Data from randomized controlled trials indicate that Riluzole can typically extend survival by 2–3 months and increase the chance of 1 more year of survival by 9% [114], underscoring its significant yet moderate neuroprotective effect. In phase II clinical studies, Nav blockers and the antiarrhythmic drug Lorcainide were tested based on a similar principle. However, due to the study’s short duration and the relatively small sample size, the persuasive power of these results remains relatively limited. Minocycline, an FDA-approved anti-inflammatory drug, has demonstrated effectiveness in the mouse models of ALS and HD. Antonenko et al. found that Minocycline primarily targets mitochondria. The impact of Minocycline on mitochondria may not be directly related to the inhibition of Ca^2+^-induced MPT (mitochondrial permeability transition) but instead to its ability to chelate Ca^2+^, bind to the RLM (rat liver mitochondria) membrane, partially uncouple the mitochondria by forming an ion channel, and ultimately prevent Ca^2+^ from accumulating in the mitochondrial matrix [115]. Metman et al. suggested that NMDA receptors’ hypersensitivity may be involved in the clinical expression of chorea-like dyskinesia in HD, and selective antagonists at this site (amantadine) can safely provide the benefits of palliative care [116]. J. Egea et al. discovered that dimebon and memantine can reduce glutamate-induced hippocampal neuron [Ca^2+^]_c_ (intracellular calcium ion) signals, where dimebon may play an indirect role in ROS generation by stabilizing mitochondria [117]. Glutamate overstimulation of its receptors can lead to abnormally elevated intracellular calcium ion concentrations, which can cause a number of processes including cellular damage and death. And when glutamate is reduced, it may interfere with the normal regulation of calcium ions by nerve cells and may instead lead to abnormally high intracellular calcium ion concentrations, a condition that can be termed excitotoxic calcium overload [118]. The excitotoxic calcium overload caused by reducing glutamate can also explain the neuroprotective effect of metformin on ischemic injury: by reducing calcium inward flow through modulation of NMDA and AMPA receptor activity [117]. Al-Izki et al.’s experiments showed that CFM6104 (a novel central nervous system-excluded sodium channel blocker) inhibited microglial activity and neural sodium loading dramatically and reduced the pace of nerve deletion accumulation and impairment in experimental autoimmune encephalomyelitis [119]. Lidster et al. showed that the systemic application of sodium ion channel blockers (oxcarbazepine) may inhibit optic neuritis in multiple sclerosis models, preventing inflammatory damage to the axons of the optic nerve and the subsequent loss of retinal ganglion cells [118]. Asseyer S et al. demonstrated that oxcarbazepine and carbamazepine act as the blockers of voltage-gated sodium channels decreasing neuronal excitability in MS [120]. Conversely, Boyle Y et al. concluded that dalfampridine is used as a VGKC antagonist to assist MS patients in motor function and has a wide range of action on subtypes Kv1.1 to 1.7, Kv2.1 and 2.2, and so on [121] (Table 3).

## 4. Conclusions and Outlook

In this study, various ion channels that can cause neurodegenerative changes are summarized. These mechanisms include the death of mitochondria, calcium overload, neuronal overexcitation, modifications in membrane ion permeability, the progressive loss of neuronal discharge, and the disruption of the myelination process. While the specific mechanisms of certain channels in regulating diseases are not yet clear, the observed changes in the channels and ions at the disease characterization level can form the basis for disease treatment and the further exploration of mechanisms. Although no targeted nor specific drugs have reached the clinical stage, numerous studies have been carried out on the more effective remission and treatment of neurodegenerative diseases at the animal level. It is believed that with in-depth research on disease mechanisms, continuous experimentation, and exploration, the classification of neurodegenerative diseases as treatable diseases is within reach. This paper discusses the influence of ion channels on neurodegenerative diseases and highlights the significance of ion channels as influencing factors in these diseases. However, there are still some limitations of this paper, which focuses on research papers in the field in recent years and lacks clinical evidence and patented inventions, which we need to be aware of and understand. In a word, the calculated use of channel modulators to specifically regulate abnormal ion channels can provide a targeted solution to channel-related problems. Revealing the regulatory mechanisms of these channels in neurodegenerative diseases can offer a more innovative treatment strategy for these conditions.

## Figures and Tables

**Table 1:** Ion channel drugs in AD.

Name	Diseases	Specific Targets	Limitations	References
Zinc (Zn^2+^)	AD	Amyloid channels	Complex intracellular regulatory mechanisms, multifunctional physiological roles, and technical challenges in clinical applications	[100]
Tetrahydrohyperforin (IDN5706)		TRPC channels	Poor specificity, safety concerns as a semi-synthetic derivative, and insufficient clinical data	[101]
Methyllycaconitine, α-bungarotoxin, and mecamylamine		nAChRs	Problems with blood–brain barrier permeability, safety, and side effects	[102]
Icariin		VGCCs	Variability in efficacy across samples, limited clinical evidence, and specific targets and pathways only	[103]
Dihydropyridine and benzothiazide		VDCCs and MNCX	Unknown side effects and safety	[104]

TRPC channels: transient receptor potential canonical channels, nAChRs: neuronal nicotinic acetylcholine receptor channels, VGCCs: voltage-gated calcium channels, VDCCs: voltage-dependent calcium channels, MNCX: mitochondrial Na^+^/Ca^2+^ exchanger.

**Table 2:** Ion channel drugs in PD.

Name	Diseases	Specific Targets	Limitations	References
Black tea extract and rosmarinic acid	PD	Mitochondrial ion channels	Lack of experimental and clinical data on human samples	[108]
Nicotine		nAChRs	Addictive and potential side effects and lack of clinical studies	[110]
Nifedipine and nimodipine		VGCCs	Individual variability and side effects	[111]
Iptakalim		Mitochondrial ATP-sensitive potassium channels	Inadequate clinical effectiveness and safety and unclear mechanism of action	[112]

nAChRs: neuronal nicotinic acetylcholine receptor channels, VGCCs: voltage-gated calcium channels.

**Table 3:** Ion channel drugs in ALS, HD, and MS.

Name	Diseases	Specific Targets	Limitations	References
Riluzole	ALS	VGSCs	Limited therapeutic efficacy, unclear mechanisms, and high prices	[113]
Minocycline	ALS and HD	Mitochondrial ion channels (mainly Ca^2+^ channels)	Safety issues and discrepancies with clinical applications	[115]
Amantadine	HD	NMDA receptors	Limited efficacy, limited research data, and unknown safety profile	[116]
Dimebon and memantine	HD	NMDA receptors	Limited efficacy and insufficient research data	[117]
CFM6104	MS	VGSCs	Unknown side effects and safety	[119]
Oxcarbazepine		VGSCs	Oxcarbazepine, as an antiepileptic drug, may primarily target specific symptoms in the treatment of MS rather than the overall course of MS	[118]
Carbamazepine		VGSCs	Risk of worsening symptoms and symptom-specific only	[120]
Dalfampridine		VGKCs	Regional variability and side effects	[121]

VGSCs: voltage-gated sodium channels, NMDA receptors: N-methyl-D-aspartate receptors, VGKCs: voltage-gated potassium channels.
